# Feminine Appeals on Cigarette Packs Sold in 14 Countries

**DOI:** 10.3389/ijph.2021.1604027

**Published:** 2021-06-14

**Authors:** Lauren Czaplicki, Kevin Welding, Joanna E. Cohen, Katherine Clegg Smith

**Affiliations:** Department of Health, Behavior, and Society, Institute for Global Tobacco Control, Johns Hopkins Bloomberg School of Public Health, Baltimore, MD, United States

**Keywords:** marketing, smoking, women, tobacco control, global health, low-and middle-income countries, tobacco packaging

## Abstract

**Objective:** Limited research has examined feminine marketing appeals on cigarette packs in low-and middle-income countries (LMICs). We reviewed a systematically collected sample of cigarette packs sold across 14 LMICs in 2013 (Wave 1) and 2015–2017 (Wave 2).

**Methods:** Packs in Wave 1 (*n* = 3,240) and Wave 2 (*n* = 2,336) were coded for feminine imagery and descriptors (flowers, fashion, women/girls, color “pink”). We examined trends in feminine appeals over time, including co-occurrence with other pack features (slim or lipstick shape, flavor, reduced harm, and reduced odor claims).

**Results:** The proportion of unique feminine cigarette packs significantly decreased from 8.6% (*n* = 278) in Wave 1 to 5.9% (*n* = 137) in Wave 2 (*p* < 0.001). Among all feminine packs, flower-and fashion-related features were most common; a substantial proportion also used flavor and reduced odor appeals.

**Conclusion:** While there was a notable presence of feminine packs, the decline observed may reflect global trends toward marketing gender-neutral cigarettes to women and a general contempt for using traditional femininity to market products directly to women. Plain packaging standards may reduce the influence of branding on smoking among women.

## Introduction

The cigarette pack is an important marketing tool for the tobacco industry and has grown in relevance as companies face increasing advertising restrictions in other mediums [1–4]. Pack shape, color, and design all communicate a brand’s “personality” to attract consumer attention [1, 4]. The use of specific colors, imagery, and text descriptors (e.g. light, mild, smooth) are especially important as they can influence perceptions of product taste and strength [5, 6], as well as the health risks associated with use [6–10]. Tobacco companies intentionally tailor packaging features to appeal to specific consumer subgroups based on demographic and lifestyle factors [11–14]. Importantly, the pack functions as a major advertising platform to draw in and recruit new smokers, including women and girls who rated feminine branded packs, such as the bright pink and black Camel No. 9 pack, as more appealing than non-feminine or non-branded, plain packs [15, 16].

The tobacco industry has long marketed smoking as a socially acceptable activity for women in high-income, Western countries like the United States, Great Britain, and Spain [17–22]. Starting in the 1920s, advertising campaigns in women’s lifestyle magazines promoted cigarettes using themes of independence, fashion, and thinness, (e.g. “Reach for a Lucky instead of a sweet”) [21–23], and the use of the cigarette as a symbol of glamour, femininity, and women’s emancipation persisted well throughout the late 20th century [22–24]. With respect to packaging, the print advertising campaigns worked in concert with the design of the cigarette and the cigarette pack itself. Tobacco companies extensively researched women’s personal and social preferences related to smoking [4, 20]. The cigarette pack often carried over the Western, stereotypical feminine appeals in the form of imagery, (e.g. flowers, butterflies [22, 25, 26]) and colors, (e.g. pastels, pink [22, 24]) of the advertising campaign, and slim or ultra-thin cigarettes were designed to reinforce the perception of smoking as a feminine, graceful, stylish activity that aligned with the idealized thin and glamourous images of women promoted in magazine ads [20, 22, 25]. Additionally, both packaging and advertisements offered different technologies that met and fortified the odor and taste preferences of women: reduced odor side-stream smoke, a light or mild cigarette that was smoother to smoke and appeared as a “healthier option,” and improved flavor through the use of menthol, mint and other flavor constituents [4, 20].

Collectively, these marketing strategies were effective at increasing cigarette sales and smoking rates among women [27]. Although women still smoke at a lower rate compared to men in nearly all countries [28], women and girls remain a potential market for long-term growth among the major transnational tobacco companies [26, 29–31]. This is particularly relevant in low-and middle-income countries (LMICs) where shifting norms around the acceptability of women smoking and increased spending power among women may facilitate smoking uptake [22, 29, 32, 33]. In 2018, less than 5% of women living in LMICs used combustible tobacco compared to 17.9% of women living in high-income countries [28]. However, in those LMICs with the highest global burden of tobacco use, rates of smoking among women is more variable. For example, while less than 3% of women in Bangladesh, China, Egypt, Pakistan, India, Indonesia, Pakistan, and Thailand smoked in 2018, the smoking rate among women in Mexico (6.5%), the Philippines (7.0%), Brazil (9.5%), Ukraine (9.9%), Russia (15.7%), and Turkey (17.0%) were much higher and some approached smoking rates among women in higher-income countries like the United States (16.7%) [28].

Further, evidence suggests that major transnational tobacco companies are employing similar marketing strategies previously used in high-income countries to target women in LMICs [22, 30, 33]. For example, cigarette advertising campaigns in countries like India and the Philippines frequently featured fashionably dressed women who conformed to Western style and beauty standards which may associate smoking with aspirations of glamour, sophistication, and independence from traditional economic and social roles held by women in LMICs [22, 26, 30, 33–35]. In addition, slim cigarettes, lipstick pack shaped cigarettes, and “light” or “mild” cigarettes have also been introduced into the marketplace of several LMCIs [22, 26, 35, 36]. Slim cigarettes that appear to be designed for women were associated with increased odds of experimental smoking among adolescents in China [35], and experimental studies conducted among young women in Mexico (16–18 years old) and Brazil (16–26 years old) found that participants rated flavored cigarette packs branded with feminine colors, (e.g. light pastels, pink) as more appealing than flavored, non-branded plain packs [36, 37]. However, little is known about the extent to which cigarette packs sold in LMICs feature feminine imagery or appeals outside of pack shape and reduced harm claims. Only a small number of studies have examined marketing features present on cigarette packs sold in LMICs [38–42], and none to date has systematically explored the presence of feminine appeals.

The current study addresses this gap and utilizes a large dataset of cigarette packs collected across 14 LMICs to examine the presence of feminine marketing appeals on packs sold in LMICs. Feminine appeals were initially informed by stereotypical, Western features, (e.g. color, fashion appeals, flowers, butterflies, and imagery of women) in advertising and pack design discussed in the extant literature and then refined based on input from country-specific experts. We document trends in the use of these overt feminine marketing appeals on packs over time and by multinational tobacco company, as well as the co-occurrence of feminine appeals with other marketing features historically used to appeal to women, (e.g. slim pack shape, flavor, “light” cigarettes, reduce odor claims). Given the role of the cigarette pack as a marketing platform to attract women and girls and increase interest in product use [15, 16, 24, 35], results from this study can elucidate the ways in which women in LMICs may be targeted by tobacco companies through cigarette packaging and inform policy strategies to reduce the impact of this marketing tactic.

## Methods

This study is part of the larger Tobacco Pack Surveillance System (TPackSS) project to monitor compliance with health warning label requirements on tobacco packages sold in 14 LMICs with the highest number of smokers at the time of initial data collection [43]. The primary aim of the TPackSS project is to purchase a comprehensive collection of unique tobacco packs sold in each TPackSS country and assess whether tobacco health warning requirements are implemented as intended. Data have been published on overall compliance with warning label requirements [44], as well as pack features that may detract from the effectiveness of warning label placement on packages, (e.g. tax stamps covering warnings) [45]. Data have also been used to explore the different marketing appeals, (e.g. English language, sports imagery, organic/natural descriptors, reduced harm terminology) present on tobacco packs across the study countries [38, 39, 41, 42].

TPackSS data collection occurred in two waves. In Wave 1 (2013), data collectors purchased cigarette packs (*n* = 3,240) from 14 LMICs: Bangladesh, Brazil, China, Egypt, India, Indonesia, Mexico, Pakistan, Philippines, Russia, Thailand, Turkey, Ukraine and Vietnam. In Wave 2 (2015–2017), data collectors purchased cigarette packs (*n* = 2,336) sold in nine of the original 14 LMICs where health warning label requirements were updated since Wave 1: Indonesia, Russia, Thailand, and Vietnam (packs purchased in 2015); Bangladesh, Brazil, India, and the Philippines (packs purchased in 2016); and China (packs purchased in 2017). Wave 2 data were not collected from Egypt, Mexico, Pakistan, Turkey, and Ukraine. Unlike the other nine countries where health warning label requirements were updated and required a new wave of compliance assessment, the warning label requirements in these five countries remained the same between 2013 and 2016 [46]. Therefore, a new compliance assessment was not required and Wave 2 data were not collected.

### Study Sample

In each LMIC, data collection occurred in select low, middle, and high socioeconomic status neighborhoods within three of the country’s 10 most populous cities. In China (five) and Wave 2 India (four), we included more than three cities in the sampling frame based on the recommendation of tobacco control expert advisors knowledgeable about the tobacco market in each country [43]. For each identified city, in-country collaborators created a sampling frame of neighborhoods by socioeconomic strata using a variety of local and national sources, including census and property value data. We then selected four neighborhoods within each socioeconomic stratum that were diverse in terms of geographic locale and residential composition. Data collectors visited a total of 12 neighborhoods in each city or 36 neighborhoods in each LMIC, except China and Wave 2 India where we included 60 and 48 neighborhoods, respectively.

Data collectors followed the same standardized protocol to systematically purchase tobacco packs across waves and within each LMIC. A detailed explanation of data collector training and the TPackSS methodology can be found in Smith et al., 2015 [43]. In brief, TPackSS staff traveled to each country to conduct a 5-day in-person training and were available during the entire data collection period to trouble-shoot issues in the field. Data collection targeted pre-selected vendor types in urban neighborhoods that were popular in each country. Packs were purchased from the most popular vendor types in each country and included tobacco shops, kiosks, supermarkets, convenience stores, stalls, street vendors, and superstores. The first vendor in each country served as the index vendor for all other stores visited in that LMIC: data collectors visited a large tobacco vendor in the first sample city, purchased all unique tobacco packs sold at the first vendor, and took a photo of the front panel of each pack to create an image archive. Field staff then visited up to five vendors in the remaining neighborhoods to identify and purchase new, unique packs not already present in the image archive. The image archive was updated following each round of new pack purchase at a vendor. Packs were considered unique if there was at least one exterior difference in the pack design, (e.g. pack size, brand name presentation, colors, promotional item, cellophane, etc.) from other packs in the image archive. Packs that looked exactly the same but had different warning label were not considered unique.

### Coding for General Pack Features

All physical packs were sent to Baltimore, Maryland United States and coded by two independent coders for a variety of marketing features. Detailed codebooks for each wave of data collection are available on the TPackSS website (https://globaltobaccocontrol.org/tpackss/resources). For the current analysis, we coded packs for features previously associated with targeted marketing to women [4, 20, 22]: slim pack shape (width of pack ≤1.3 cm), lipstick pack shapes (tall, slender rectangular pack), and flavored packs based on the presence of text (e.g., mint), imagery, (e.g. mint leaf), or flavor capsules which release a flavored liquid into the filter when pressed. Flavor categories included fruit or citrus, alcoholic/energy drink, menthol/mint, clove kretek, other characterizing flavor, other non-characterizing flavor, and unknown flavor capsule. Other characterizing flavors included coffee/tea-, dessert-, herbal-, incense-, and spice-related flavors. Non-characterizing flavors, on the other hand, included “concept” flavors that did not belong to a traditional characterizing flavor category, (e.g. menthol, fruit, dessert, etc.) but rather connoted a taste, smell, or sensory experience using descriptive terms such as “fresh,” “ice burst,” and “purple.” The unknown flavor capsule category included those packs with capsule technology as indicated by the pack or stick branding but no other text or imagery to indicate flavor type. Additionally, we included claims related to reduced odor and smell or reduced harm (“light/lights”; “mild/low” cigarettes).

To capture the global circulation and ownership of feminine appealing packs, we also categorized whether the pack was illicitly sold in the country of purchase (yes/no) and the tobacco company that manufactured each pack. We identified packs as illicit if the health warning label required by the country where the pack was purchased was not present on the pack. For tobacco company, we used a combination of the manufacturing information printed on the pack itself and information on the company structure to identify the tobacco company. In this analysis, we present results for the major tobacco companies (British American Tobacco, China National Tobacco Company, Imperial Tobacco Company, Japan Tobacco International, Korea Tobacco and Ginseng, Philip Morris International) at the time of data collection. We grouped known subsidiary companies together, (e.g. Philip Morris Thailand LTD., Philip Morris Mexico, or Philip Morris Ukraine) with their parent company to create one group per company.

### Feminine Appeals Coding

Packs were also coded for the presence of overt feminine marketing appeals discussed in the existing literature [15, 16, 20–26, 30, 35–37]. Specifically, we coded packs as feminine if they contained imagery or descriptors associated with the following: flowers/butterflies, fashion, (e.g. images of jewelry, the term “stylish,” animal prints), women/girls, (e.g. non-sexualized images of women/girls, terms like “lady” or “girl”), the word “pink” or the color pink, and other potential feminine cues, including imagery or descriptors associated with concepts like hearts, kisses, and romance. Brand names, (e.g. *Vogue* or *Glamour*) were excluded from text-based coding to ensure that other feminine features beyond brand name were present on the pack to include it in the sample. We also included all lipstick shaped packs in our initial sample of feminine packs. We did not include all slim shaped packs in the initial dataset because, unlike lipstick shaped packs which are almost exclusively associated with women, slim packs could have a broader appeal across gender [36, 47].

Our coding categories and decisions were heavily informed by stereotypical Western concepts of femininity based on the literature, which may not always communicate femininity in other countries. To ensure that coding was culturally relevant, we identified tobacco control experts from each LMIC and asked them to assess whether packs in our initial sample were feminine based on their knowledge of country-specific norms and feminine symbols. Expert coders also provided a brief rationale for their decision. We next compared the expert coding against the initial coding. In our initial coding, we categorized 656 packs as feminine. In nearly all cases where the expert coder disagreed with the initial coding (*n* = 241 packs), we accepted the expert coder’s decision and rationale for why the pack was not feminine. In the few cases of disagreement where the expert rationale was unclear or missing (*n* = 5), we discussed the pack with the expert and arrived at a final coding decision. Our final sample included 415 feminine packs. Table 1 provides a descriptive narrative of the type of feminine appeals included the study sample by country.

**TABLE 1:** Descriptive narrative of overt feminine appeals identified by expert coders from 14 low- and middle-income countries (Bangladesh, Brazil, China, Egypt, India, Indonesia, Mexico, Pakistan, Philippines, Russian Federation, Thailand, Turkey, Ukraine, Vietnam) included in the Tobacco Pack Surveillance System from 2013 to 2017.

Country	Narrative of feminine concepts discussed by in-country expert coders
Bangladesh	Feminine appeals included imagery of flowers, pink colors, a graphic of famous actress Marilyn Monroe, and rhinestone jewelry. Textual cues included phrases like “queen edition.” Slim and lipstick shaped packs with these features were also considered feminine by the expert coder, but slim packs with blue colors were not considered feminine
Brazil	Feminine appeals included imagery of flowers and pink colors. Textual cues included phrases like “designed in Paris.” Slim and lipstick shaped packs with these features were considered feminine by the expert coder. Packs that included text such as “style” or “trend” to describe the design or variant of the product without any of other feminine features were not considered feminine
China	Feminine appeals included imagery of flowers but only when the cultural meaning of the flower aligned with femininity, (e.g. peonies may represent wealth or success and were not considered feminine, while hibiscus signifies beauty and romance and were considered feminine). Other imagery included pink colors, emoji-like feminine figures, hearts, and diamonds. Textual cues included “twelve beauties of Jinlin.” Slim and lipstick shaped packs with these features were considered feminine by the expert coder. Textual cues like “cool fashion” without any other feminine indicator were not considered feminine
Egypt	Feminine appeals included imagery of flowers, pink colors, and colorful circular design. Slim packs with these features were considered feminine. Packs that included text such as “style” to describe the cigarette design without any other feminine features were not considered feminine. In addition, imagery of Queen Cleopatra was considered a symbol of Egypt’s heritage rather than feminine imagery by the expert coder
India	Feminine appeals included pink colors and the presence of textual cues like “stylish” on a mauve background. Slim packs with these features were considered feminine. Imagery of hearts or the silhouette of a woman dancing with a man in a celebratory scene were not considered feminine by the expert coder
Indonesia	Feminine appeals only included lipstick shaped packs. Packs that used a combination of flowers and tobacco plant imagery or images of women in traditional dress were not considered feminine by our expert coder
Mexico	Feminine appeals included fashionable animal print imagery and pink/purple colors. Textual cues included phrases like “I pink, therefore I am.” Lipstick shaped packs with these features were considered feminine by the expert coder
Pakistan	Feminine appeals included imagery of flowers, pink colors, and rhinestone jewelry. Textual cues include phrases like “garden romance” and “delicate scent.” Slim and lipstick shaped packs with these features were considered feminine by the expert coder, but slim packs with blue colors or reddish-brown colors were not considered feminine. In addition, imagery of the silhouette of a woman in traditional dress dancing was not considered feminine imagery by the expert coder
Philippines	Feminine appeals included pink colors and textual cues like “for a stylish leader.” Slim packs with these features were considered feminine by the expert coder
Russian federation	Feminine appeals included flowers, pink colors, and graphics of thin, fashionably dressed young women (see Figure 2). Textual cues included phrases like “romance” and quotes from fashion designers, (e.g. “to be beautiful, all a woman needs is a black pullover and a black skirt and to be arm in arm with a man she loves.”-Yves Saint Laurent). Packs that included imagery of traditionally dressed women were not considered feminine by the expert coder
Thailand	Feminine appeals included pink color. Slim packs with pink colors were considered feminine, but a slim pack with green colors was not considered feminine by the expert coder
Turkey	Feminine appeals included imagery of flowers, pink colors, and fashionable animal prints. Slim packs with these features were considered feminine, but slim packs with royal blue colors were not considered feminine by the expert coder
Ukraine	Feminine appeals included imagery of flowers, butterflies, pink colors, and fashionable animal prints. Textual cues included phrases like “eleganza,” “romance,” and “fantasy.” Slim and lipstick shaped with these features were considered feminine, but slim packs with blue, red, or black colors were not considered feminine by the expert coder. In addition, packs that included imagery of traditionally dressed women were not considered feminine by the expert coder
Vietnam	Feminine appeals included imagery of flowers, pink colors, lipstick kisses, and hearts. Textual cues included phrases like “kiss me” and “romantic.” Slim packs with these features were considered feminine, but slim packs with red, black, blue, and green colors (two of which had flowers present) were not considered feminine by the expert coder

### Statistical Analysis

We conducted Fischer’s exact tests of association to examine trends in feminine appeals over time using Stata16 software. We assessed differences by country, major tobacco company, and the presence of other pack features (pack shape, flavor, reduced harm, and reduced odor claims). Tests of association were two sided (*p* < 0.05).

## Results

Table 2 presents the proportion of cigarette packs purchased in Wave 1 (2013) and Wave 2 (2015–2017) that contained overt feminine appeals by country, tobacco company, and pack-specific attributes. Overall, the proportion of feminine cigarette packs in the study samples significantly decreased from 8.6% (*n* = 278 packs) in Wave 1 to 5.9% (*n* = 137 packs) in Wave 2 (*p* < 0.001).

**TABLE 2:** Presence of any overt feminine appeals on cigarette packs (*n* = 5,575) purchased from 14 low-and middle-income countries (Bangladesh, Brazil, China, Egypt, India, Indonesia, Mexico, Pakistan, Philippines, Russian Federation, Thailand, Turkey, Ukraine, Vietnam) included in the Tobacco Pack Surveillance System in Wave 1 (2013) and Wave 2 (2015–2017) by country and other pack attributes.

	Wave 1 (2013)		Wave 2 (2015–2017)		*p* value^b^
	Sample size	Feminine packs^a^ % (*n*)	Sample size	Feminine packs^a^ % (n)	
Overall	3,240	8.6% (278)	2,336	5.9% (137)	<0.001
Country					
Ukraine	324	21.6% (70)	–	–	–
Russian Federation	502	20.7% (104)	502	13.5% (68)	0.003
Brazil	122	8.2% (10)	147	2.0% (3)	0.023
China	453	7.3% (33)	738	7.9% (58)	0.738
Vietnam	147	5.4% (8)	150	0.0% (0)	0.003
Egypt	58	5.2% (3)	–	–	–
Pakistan	382	4.2% (16)	–	–	–
Turkey	308	2.9% (9)	–	–	–
Thailand	126	2.4% (3)	111	0.0% (0)	0.250
Bangladesh	191	2.1% (4)	233	3.0% (7)	0.761
Philippines	143	2.1% (3)	108	0.0% (0)	0.262
Mexico	134	8.2% (11)	–	–	–
India	135	1.5% (2)	95	1.0% (1)	1.000
Indonesia	215	0.9% (2)	252	0.0% (0)	0.211
Tobacco company					
British American tobacco	547	5.7% (31)	358	2.5% (9)	0.030
China National tobacco company	438	6.8% (30)	513	9.2% (47)	0.233
Imperial tobacco company	304	10.5% (32)	133	8.3% (11)	0.601
Japan Tobacco international	406	14.5% (59)	236	10.2% (24)	0.143
Korea tobacco and Ginseng	132	12.1% (16)	109	5.5% (6)	0.114
Philip Morris international	511	6.6% (34)	326	2.1% (7)	0.003
Flavor capsule cigarette^c^					
Yes	85	1.2% (1)	137	0.0% (0)	0.383
Flavor type^d^					
Any flavor	685	11.5% (79)	542	5.2% (28)	<0.001
Fruit or citrus	106	31.1% (33)	84	10.7% (9)	0.001
Alcoholic/Energy drink	34	32.3% (11)	17	11.8% (2)	0.175
Menthol/Mint	270	11.8% (32)	204	5.9% (12)	0.037
Clove/Kretek	227	1.3% (3)	191	0.0% (0)	0.254
Other characterizing flavor^e^	37	10.8% (4)	42	14.3% (6)	0.743
Other non-characterizing flavor^f^	73	4.1% (3)	75	0.0% (0)	0.117
Unknown flavor capsule^g^	26	3.8% (1)	24	0.0% (0)	1.000
Pack shape					
Slim pack^h^	512	35.9% (184)	273	25.3% (69)	0.002
Lipstick shaped pack^i^	36	100.0% (36)	7	28.6% (2)	<0.001
Claims					
“Reduced odor”	66	51.5% (34)	101	26.7% (27)	0.002
“Light/lights”descriptor	143	2.8% (4)	43	2.3% (1)	1.000
“Mild/low”descriptor	120	0.83% (1)	67	0.0% (0)	1.000
Illicit pack^j^					
Yes	772	5.6% (43)	450	3.3% (15)	0.094

a Pack includes any imagery and/or descriptors associated with flowers/butterflies, fashion, women or girls, pink, and other appeals, (e.g. hearts, lipstick kisses).

b *p* values reported for Fisher’s exact test of association (2-sided).

c Flavor capsule cigarette packs were identified by the presence of imagery or text on the cigarette stick or pack that indicated a flavor capsule was present to press and release a flavor into the filter.

d Flavor categories are not mutually exclusive and were determined based on the presence of flavor-related text, (e.g. mint), imagery, (e.g. mint leaf), and flavor capsules.

e Other characterizing flavor included coffee, tea, caramel/chocolate/vanilla, dessert/sweets, cinnamon or other spice, herbs, and incense flavors.

f Other non-characterizing flavor included “concept” flavors that did not belong to a traditional characterizing flavor category but connoted a taste, smell, or sensory experience using descriptive terms such as “fresh,” “ice burst,” and “purple.”

g Unknown flavor capsule included packs with capsule technology but no other text or imagery to indicate flavor type.

h Slim packs were considered those packs where the width of the side of the pack was 1.3 cm or less.

i Lipstick shaped packs were considered tall, slender packs with a square top panel and equal width of side panels.

j Illicit packs included packs sold without the country required health warning label present.

By country, Ukraine had the highest proportion of unique feminine cigarette packs (21.6%, *n* = 70 packs) in Wave 1 followed by Russia (20.7%, *n* = 104 packs), Brazil (8.2%, *n* = 10 packs), China (7.3%, *n* = 33 packs), and Vietnam (5.4%, *n* = 8 packs). In Wave 2, a significantly smaller proportion of packs from Russia (13.5%, *n* = 68 packs), Brazil (2.0%, *n* = 3 packs), and Vietnam (0%, *n* = 0 packs) contained overt feminine appeals (all *p*’s < 0.05). The proportion of feminine packs purchased in China remained stable over time (7.9%, *n* = 58 packs in Wave 2). There was no Wave 2 pack collection in Ukraine.

Most cigarette packs included in this study were brands owned by major multinational tobacco companies. Across waves, over 10% of packs from Japan Tobacco International contained overt feminine appeals (14.5%, *n* = 59 packs Wave 1; 10.2%, *n* = 24 packs Wave 2, *p* = 0.143). In contrast, a smaller proportion of packs from British American Tobacco (5.7%, *n* = 31 packs) and Phillip Morris International (6.6%, *n* = 34 packs) contained feminine appeals in Wave 1, and the proportion of overtly feminine packs from both tobacco companies significantly declined in Wave 2 (see Table 2). The most common brands across waves included *Vogue* from British American Tobacco (*n* = 24 packs Wave 1, *n* = 8 packs Wave 2), *Glamour* from Japan Tobacco International (*n* = 24 packs Wave 1; *n* = 14 packs Wave 2), *Kiss* from Richmond Tobacco Trading Ltd. (*n* = 27 packs Wave 1, *n* = 14 packs Wave 2), *Style* from Imperial Tobacco Company (*n* = 15 packs Wave 1, *n* = 9 packs Wave 2), and *Nanjing* from China National Tobacco Corporation (*n* = 10 packs Wave 1, *n* = 11 packs Wave 2).

In Wave 1, a larger proportion of alcoholic/energy drink flavored cigarettes (32.3%, *n* = 11 packs) followed by fruit flavored cigarettes (31.1%, *n* = 33 packs) and menthol/mint flavored cigarettes (11.8%, *n* = 32 packs) contained feminine appeals. In Wave 2, the proportion of fruit (10.7%, *n* = 9 packs) and menthol/mint flavored packs (5.8%, *n* = 12 packs) with overt feminine appeals significantly decreased (*p* = 0.001 and *p* = 0.037, respectively). With respect to pack shape, nearly 36% of slim packs (*n* = 184 packs) and all lipstick-shaped packs (100%, *n* = 36 packs) in Wave 1 contained feminine appeals. In Wave 2, a significantly smaller proportion of slim packs (25.3%, *n* = 69 packs, *p* = 0.002) and lipstick-shaped packs (28.6%, *n* = 2 packs, *p* < 0.001) contained feminine appeals.

Across waves, very few cigarette packs with flavor capsules or the use of “light/lights” or“mild/low” descriptors contained overt feminine appeals. In Wave 1, around half (51.5%, *n* = 34 packs) of cigarette packs with “reduced odor” claims contained feminine appeals; however, in Wave 2 the proportion of “reduced odor” packs with feminine appeals significantly declined (26.7%, *n* = 27 packs, *p* = 0.002).

The proportion of illicit packs sold in a country that contained overt feminine appeals was small overall and decreased from 5.6% (*n* = 43 packs) in Wave 1 to 3.3% (*n* = 15 packs, *p* = 0.094) in Wave 2.

**FIGURE 1:**
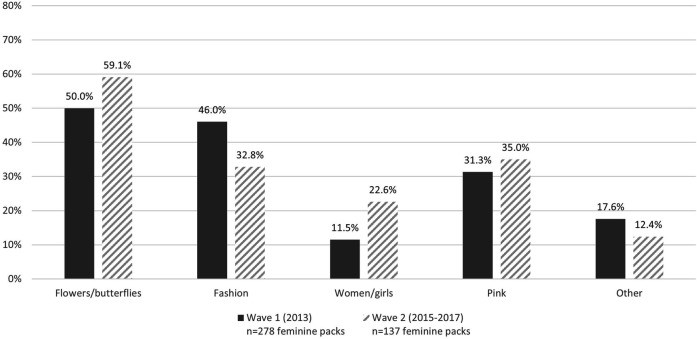
Image and descriptor-based overt feminine appeals by category among feminine cigarette packs (*n* = 415) purchased in 14 low-and middle-income countries (Bangladesh, Brazil, China, Egypt, India, Indonesia, Mexico, Pakistan, Philippines, Russian Federation, Thailand, Turkey, Ukraine, Vietnam) included in the Tobacco Pack Surveillance System in Wave 1 (2013) and Wave 2 (2015–2017).

**FIGURE 2:**
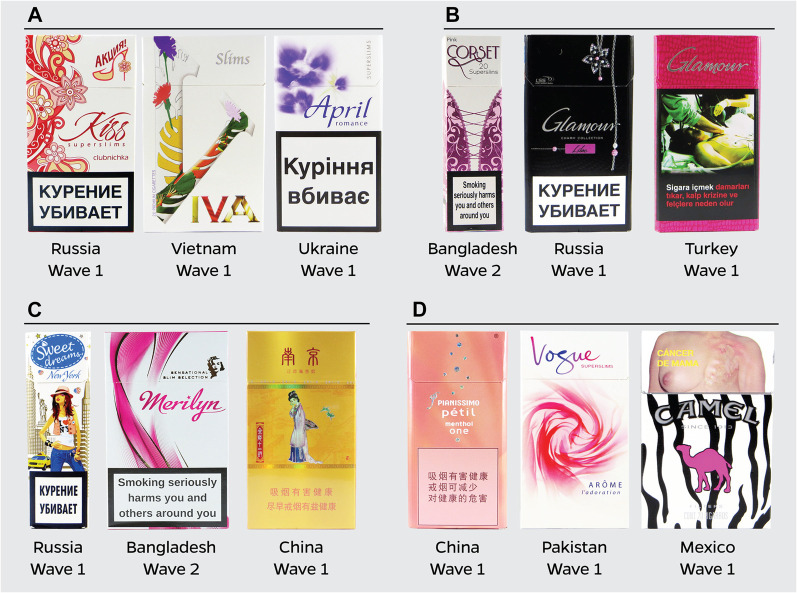
Exemplar images of feminine packs purchased across 14 low-and middle-income countries (Bangladesh, Brazil, China, Egypt, India, Indonesia, Mexico, Pakistan, Philippines, Russian Federation, Thailand, Turkey, Ukraine, Vietnam) included in the Tobacco Pack Surveillance System from 2013 to 2017 by major appeal category: **(A)** flowers/butterflies **(B)** fashion **(C)** women/girls and **(D)** the color pink.

Figure 1 displays the proportion of overtly feminine packs by type of appeal category. Flower-related imagery or descriptors were commonly featured on feminine packs, and at least 50% of feminine packs in Wave 1 and Wave 2 contained flower-based appeals. Fashion-related features were also common and present on 46.0% of feminine packs in Wave 1; however, the presence of fashion appeals significantly decreased in Wave 2 (32.8%, *p* = 0.011). While imagery and descriptors related to pink were present on approximately one-third of feminine packs in both waves, there was a significant increase in imagery or text associated with women or girls from Wave 1 (11.5%) to Wave 2 (22.6%, *p* = 0.005). Figure 2 presents examples of stereotypical feminine packs by the four main appeal categories.

## Discussion

In this study, we found that a notable number of cigarette packs sold in 14 LMICs contained overt feminine imagery or textual cues. This finding aligns with previous reports that women in LMICs are an important growth market for the tobacco industry [22, 29, 32, 33], and reinforces the role of the cigarette pack as a marketing vector to communicate a brand’s intended user [4]. Overall, transnational tobacco companies produced a substantial number of the feminine packs purchased in our study. Although we saw some differences in how femininity was characterized across countries, the feminine packs in this study largely utilized many of the same stereotypical Western marketing features, (e.g. fashion, flowers, pink) and pack features, (e.g. flavor, reduced odor, slim pack shape) that were previously employed to target to women in higher income countries [20, 22, 24]. We also found that the use of terms and imagery associated with women and girls in our sample of feminine packs increased over time, reflecting the potential importance of these marketing features in the context of LMICs. Our findings suggest that the major tobacco companies continue to draw on a consistent playbook to rely on Western standards of femininity to attract the attention and reinforce the sensory and pack shape preferences of women in LMICs [22, 26]. Smaller, domestic tobacco companies like the Richmond Tobacco Trading Ltd. also appeared to utilize the same or similar tactics as the transnational companies to market feminine packs, (e.g. *Kiss*) in their local market [22]. Collectively, these findings demonstrate efforts across tobacco companies to design packs that signal the suitability of smoking among women in LMICs and potentially increase receptivity to smoking among this demographic group through appealing branded packs [15, 16, 35–37].

One major finding from this study is that the proportion of overtly feminine packs in our systematic sample declined in almost all LMICs over time. Across both study waves, we purchased very few or no feminine packs in countries with low smoking rates among women such as Thailand, the Philippines, Indonesia, and India [28]. At the same time, the proportion of feminine packs in countries with higher rates of smoking among women, like Russia, remained relatively high but also decreased over time [28]. We are limited in our ability to fully monitor this trend given the lack of Wave 2 data from Ukraine—the country with the highest proportion of feminine packs in Wave 1. However, when Ukraine is excluded from the analysis, we still observed a similar, though not statistically significant, decrease in the proportion of feminine packs from Wave 1 (7.1%) to Wave 2 (5.9%, *p* = 0.073).

Overall, there were no changes in tobacco control policies regarding product packaging in the study countries between Wave 1 and Wave 2 that could potentially account for this decline [46]. However, one potential explanation for the decline is that fewer overtly feminine brands and brand variants were on the market from Wave 1 to Wave 2. Tobacco companies create brand variants that differ by color, pack design, and brand or product descriptors, (e.g. “Marlboro Red”; “ultra-light”) to target different groups of consumers [48, 49], and they invest heavily in market research to understand the appeal of these different brands and brand variants [14, 20, 50, 51]. It is possible that the industry only focused on their most successful brands and brand variants and stopped producing those with lower market sales or less appeal to women.

Another possible explanation for the observed decline in overtly feminine packs is that tobacco companies have either slowed or stopped efforts to target cigarettes to women using the explicit feminine cues. In some LMICs, it is possible that overtly targeting women with branded packaging is not culturally acceptable and other approaches are needed [22, 30]. For example, in India brands like *Platinum*, a cigarette that explicitly targeted women and their preference for silver jewelry, was less successful than other cigarette brands that used more subtle, indirect cues to link smoking with a woman’s sophistication and sexual allure in billboard and magazine advertising [34]. Additionally, there has been a global shift toward marketing gender-neutral (“dual-sex” [20]) cigarettes to women in Western, high-income countries [18, 20], and a general reactance against the use of traditional femininity and female independence to market products directly to women [52–55]. It may be that a similar trend toward less explicitly feminine and more gender non-specific branding and technologies (e.g., flavor capsules) that contain features that appeal to men and women equally is also taking place in the LMICs included in this study. Emerging evidence suggests that women in several LMICs are more likely than men to prefer cigarette packs with flavor capsules [56, 57], although both groups find capsule packs appealing [56, 58]. This corresponds to cross-cultural research in 10 countries, which included India, China, Russia, and Brazil, that suggests brands that have equally high levels of masculine and feminine appeals (referred to as *gender androgenous*) have greater reported brand value vs. brands that are exclusively feminine or exclusively masculine [59]. Given the increase in the number of capsule packs in our sample from Wave 1 (*n* = 85) to Wave 2 (*n* = 137), it is possible that tobacco companies are targeting women through novel and more gender androgenous or gender-non-specific capsule packs.

A notable exception to the overall decline in overtly feminine packs observed in this study may be China, where the proportion of feminine packs purchased in this study remained stable and high despite the low rates of smoking among women in China [28]. It may be more culturally appropriate in China to market cigarettes with overt feminine appeals. Further, China was the only study country with a significant change in marketing restrictions on tobacco advertising between Wave 1 and Wave 2, where tobacco advertising in public places, including retail stores, and mass media was banned, effective September 2015 [60]. It is possible that tobacco companies may continue to focus attention on marketing cigarettes to consumers through appealing pack designs given the more recent limitations on promoting cigarettes through other advertising platforms [4]. Continued surveillance to examine changes in how cigarettes are marketed to women in China is warranted, including whether there is a general shift toward more gender non-specific packaging.

Despite some slightly different conceptualizations of femininity across countries (see Table 1), feminine packs were identified in every country included in this study. These findings can be useful for tobacco control efforts, primarily policies to reduce branding/targeting of cigarette packs such as single presentation requirements and plain packaging. A single presentation policy, like the one adopted in Uruguay in 2009, could reduce the number of brand variants on the market to only one, single variant per brand family and limit any proliferation of packs aimed at women [49]. Plain packaging policies like those recently implemented in study countries Thailand (2019) and Turkey (2020) could remove branded packaging altogether and require all brands be sold in packs with the same standard color, font, and layout [61]. Both policy options—alone or in combination [48, 49]—have the potential to reduce the influence of the pack itself as an avenue to increase product appeal among consumers, including women in LMICs [36, 37].

This study is subject to several limitations. First, data were derived from a select number of LMICs where the global burden of tobacco use is the highest and our findings may not generalize to other LMICs, particularly in under-represented regions like Africa. Given the design of the TPackSS study to assess compliance with new health warning label requirements over time, we are also limited in our ability to examine trends in feminine marketing in some countries, like Ukraine, where no change in warning label rules occurred between waves and Wave 2 data were not collected. Second, we only purchased cigarette packs from the most populous cities in each country. It is possible that cigarette packs not collected in this study were available in smaller cities and rural areas of a country, which would affect our estimate of the proportion of unique overtly feminine packs in a sold in a country. Additionally, our data collection is intended to capture the breadth of packs available, therefore, the proportions presented are not weighted to reflect the relative market share or popularity of each brand or brand variant. Future analyses of brand-level sales data could complement our study and offer insight into the patterns of sales of feminine packs over time. Finally, our coding structure for overt feminine appeals may not have captured all images or descriptors stereotypically associated with femininity. Even though we worked with an expert from each country to ensure the cultural relevance of our predetermined coding categories, it is possible that we underreport the prevalence of feminine packs across countries if specific feminine imagery or terms were not accounted for in our codebook or in the expert review.

### Conclusion

Prior research highlights the importance of women in LMICs as a potential growth market for the tobacco industry [26, 29–31], and the cigarette pack is one key platform to target women in LMICs, particularly as more countries implement bans on advertising and promotion through other channels [46]. Our study found that both transnational and domestic tobacco companies use stereotypical feminine imagery and text to market cigarette packs to women in LMICs. Although we observed an overall decline in the proportion of overtly feminine packs sold in select LMICs over time, our results indicate that imagery or descriptors related to flowers, the color pink, and women and girls remain relevant features on feminine packs. The decline observed may reflect global trends toward marketing gender non-specific cigarettes to women and a general contempt for using traditional femininity to market products directly to women. Plain and standardized packaging can potentially reduce the influence of branded cigarette packs on increasing product appeal, including among women [15, 16, 36, 37]. Importantly, such policies could potentially limit exposure to pack branding for other groups such as youth or low-income populations [38, 62], that may also be disproportionately targeted by cigarette pack marketing.
